# Progressive changes in microglia and macrophages in spinal cord and peripheral nerve in the transgenic rat model of amyotrophic lateral sclerosis

**DOI:** 10.1186/1742-2094-7-8

**Published:** 2010-01-28

**Authors:** David J Graber, William F Hickey, Brent T Harris

**Affiliations:** 1 Department of Pathology, Dartmouth Medical School, One Medical Center Drive, Lebanon, New Hampshire 03756, USA

## Abstract

**Background:**

The role of neuroinflammation in motor neuron death of amyotrophic lateral sclerosis (ALS) is unclear. The human mutant superoxide dismutase-1 (hmSOD1)-expressing murine transgenic model of ALS has provided some insight into changes in microglia activity during disease progression. The purpose of this study was to gain further knowledge by characterizing the immunological changes during disease progression in the spinal cord and peripheral nerve using the more recently developed hmSOD1 rat transgenic model of ALS.

**Methods:**

Using immunohistochemistry, the extent and intensity of tissue CD11b expression in spinal cord, lumbar nerve roots, and sciatic nerve were evaluated in hmSOD1 rats that were pre-clinical, at clinical onset, and near disease end-stage. Changes in CD11b expression were compared to the detection of MHC class II and CD68 microglial activation markers in the ventral horn of the spinal cord, as well as to the changes in astrocytic GFAP expression.

**Results:**

Our study reveals an accumulation of microglia/macrophages both in the spinal cord and peripheral nerve prior to clinical onset based on CD11b tissue expression. The microglia formed focal aggregates in the ventral horn and became more widespread as the disease progressed. Hypertrophic astrocytes were not prominent in the ventral horn until after clinical onset, and the enhancement of GFAP did not have a strong correlation to increased CD11b expression. Detection of MHC class II and CD68 expression was found in the ventral horn only after clinical onset. The macrophages in the ventral nerve root and sciatic nerve of hmSOD1 rats were observed encircling axons.

**Conclusions:**

These findings describe for the first time in the hmSOD1 rat transgenic model of ALS that enhancement of microglia/macrophage activity occurs pre-clinically both in the peripheral nerve and in the spinal cord. CD11b expression is shown to be a superior indicator for early immunological changes compared to other microglia activation markers and astrogliosis. Furthermore, we suggest that the early activity of microglia/macrophages is involved in the early phase of motor neuron degeneration and propose that studies involving immunomodulation in hmSOD1transgenic models need to consider effects on macrophages in peripheral nerves as well as to microglia in the spinal cord.

## Background

In addition to motor neuron loss and paralysis, evidence of reactive microglia/macrophages and astrocytes is observed in motor regions of the CNS in sporadic and familial amyotrophic lateral sclerosis (ALS) [1,2], as well as in the ALS animal models of transgenic mice and rats expressing human mutant SOD1 (hmSOD1) [3-7]. The role of the microglia and/or infiltrating macrophages in motor neuron degeneration has been difficult to ascertain and is the focus of several reviews [8,9]. Although administration of immunosuppressive drugs early in disease development has extended survival in the transgenic mouse model of ALS [10-12], such treatment has not been successful in clinical trials for ALS to date [13-17].

Increased gene expression for several cytokines has been identified early in disease development in spinal cord tissues from hmSOD1 transgenic mice and rats [18-21]. Onset and progression of reactive microglia or infiltrating macrophages have also been reported previously. Microglia identified with CD11b, a constitutive marker of myeloid cells, are increased at clinical onset and increase further by disease end-stage in the hmSOD1 murine model [6,22]. The extent of CD11b expression is also elevated in the hmSOD1 rat model even prior to clinical onset [5]. Cells expressing MHC class II occur after clinical onset in hmSOD1 mice [3], whereas induction of CD68 expression has been reported pre-clinically [23]. The onset of various microglial activation markers has not been fully explored in the rat model or altogether throughout disease progression.

Although gliosis is well documented along with neurodegeneration in the CNS, motor axons in the peripheral nervous system (PNS) are also lost early in disease development [24-26]. Accumulation of macrophages in sciatic nerve and ventral nerve root has been described when the murine hmSOD1 model was initially developed [25], but little is known regarding their progression or their occurrence in the rat model.

Using the hmSOD1 transgenic rat model of ALS, we investigate the progression of reactive microglia in the spinal cord and macrophage activity in the PNS. We describe for the first time in the rat model that in addition to the early enhancement of microglial CD11b expression in the ventral horn, macrophages accumulate in the ventral nerve root and sciatic nerve pre-clinically. Also, astrogliosis and other microglia activation markers (MHC class II and CD68) occur in the ventral horn later in disease development relative to the enhancement of CD11b expression.

## Methods

### Animals

Hemizygous hmSOD1 (G93A, L26H line) rats on a Sprague-Dawley background were obtained from Taconic Farms (Germantown, NY). The Institutional Animal Care and Use Committee at Dartmouth College approved all experimental protocols. The colony was subsequently maintained by breeding hmSOD1 male rats with wild-type Sprague-Dawley females. About one half of the offspring carried the SOD1 mutation; they were identified at three weeks of age by genotyping for human SOD1 with tissue from ear punches that was digested overnight at 55°C in 200 μl DirectPCR lysis reagent (Viagen Biotech Inc., Los Angeles, CA) containing 2 mg/ml of proteinase K (Roche Diagnostics, Inianapolis, IN). Solutions were vortexed, heated at 85°C for 45 min, and 1 μl was added to a PCR mastermix containing water (9.5 μl), 10× buffer (2 μl), Q (4 μl), dNTP (0.4 μl), 20 μM forward and reverse primers (1 μl each), and TAQ (1 μl). Positive bands were detected with ethidium bromide on 1% agarose electrophoresis gels that were run with confirmed positive and negative hmSOD1 controls. Rat GAPDH primer served as a control.

Animals were allowed to feed *ad libitum *with two or three rats per cage, and both water and food were always within reach of animals through to end-stage of disease. Weighing and scoring of motor deficits were performed by an examiner blinded to the genotype of the animals. Weights were recorded twice per week before 90 days of age and then four times per week thereafter. Gait and limb paralysis was assessed for all animals during weighing sessions and each limb was recorded as having paresis (limping with limited range of motion) or paralysis (no movement). Each rat was assigned a score based on the number and state of affected limbs as shown in Table 1 (unpublished scoring developed by D. Howland, Wyeth Research, Princeton, New Jersey). Tissues were collected from hmSOD1 rats and assessed based on age or categorized into four disease stages (Table 2): 1) early pre-clinical; 2) late pre-clinical; 3) clinical onset (normal gait, but stopped gaining weight) 4) disease end-stage (limb paralysis and 20% loss in maximum weight ). Tissues from age-matched wild-type littermates were also collected.

**Table 1:** Limb paralysis scoring of hmSOD1 rats

score	Number of Affected Limbs	Limb Phenotype
**8**	0	normal gait and range of motion
**7**	1	paresis
**6**	1	paralysis
	2	paresis
**5**	2	1 paresis, 1 paralysis
	3	paresis
**4**	2	paralysis
	3	2 paresis, 1 paralysis
**3**	3	1 paresis, 2 paralysis
	4	3 paresis, 1 paralysis
**2**	3	paralysis
	4	2 paresis, 2 paralysis
**1**	4	1 paresis, 3 paralysis
**0**	4	paralysis

Scoring criteria of motor impairment in hmSOD1 rats based on detection of limb paresis (limited movement) and paralysis (no movement).

**Table 2:** Details of hmSOD1 rats per disease stages

Disease Stage	Number of Animals (male, female)	Average Age in Days +/- SD	Average Recent Weight Change* in g/day +/- SD	Average Motor Score** +/- SD
**Early Pre-Clinical**	4 (3,1)	70.5 +/- 6.6	3.2 +/- 1.5	8
**Late Pre-Clinical**	5 (4,1)	90.8 +/- 1.6	2.7 +/- 0.9	8
**Clinical Onset**	5 (4,1)	105.2 +/- 5.0	-0.3 +/- 1.0	8
**End-Stage**	6 (4,2)	119.5 +/- 5.5	-10.0 +/- 3.9	3.5 +/- 1.0

Number, age, and clinical phenotype of hmSOD1 rats in each disease stage that were used for histological analysis. SD represents standard deviation. *Recent Weight change was recorded over a 10 day period. **See Table 1 for criteria of motor scoring.

### Immunohistochemistry

Cervical-thoracic and lumbar segments of spinal cord that were approximately 2 cm in length and both sciatic nerves were rapidly dissected from hmSOD1 and wild-type rats ranging from 60 to 130 days of age following CO_2 _euthanasia. Spinal cords were prepared for immunohistochemistry by two methods. (1) Fresh-frozen tissues were submerged in OCT-embedding medium (Sakura, Tokyo, Japan) for 5 min, then snap-frozen and stored at -80°C. (2) Paraformaldehyde-immersed tissues were immediately fixed in 4% buffered paraformaldehyde for 2 h at 4°C, rinsed with PBS, and stored overnight in 30% sucrose solution. These tissues were then immersed in room temperature OCT for 15 min, then rapidly frozen, and stored at -80°C. Spinal cords segments from similarly aged hmSOD1 and wild-type rats were embedded within the same OCT sectioning block. Seven-micron thick transverse-sections were cut with a cryostat microtome (Leica Microsystems, Deerfield, IL) and mounted on Superfrost/Plus microscope slides (Thermo Fisher Scientific, Waltham, MA). Fresh-frozen sections were immediately fixed in cryostat temperature methanol for one minute and subsequent steps were performed in 0.5 M Tris-HCl buffer. Paraformaldehyde -immersed sections involved no further fixation and were performed in 0.1 M PBS. Blocking and subsequent antibody solutions contained 1% bovine serum albumin and 1% normal serum from goat or fetal bovine. Sections were treated with primary antibodies overnight at 4°C. Tissue sections without primary antibody were used to control for background staining.

Microglia and macrophages were labeled with mouse anti-CD11b (OX-42 supernatant). The OX-42 antibody to rat CD11b is superior relative to antibodies to murine CD11b. Rabbit anti-Iba1 (1:650; Wako Chemicals USA, Richmond, VA), mouse anti-CD68 (ED1; 1:300; Serotec, Raleigh, NC), and MHC class II (OX-6 supernatant) were also used to label microglia/macrophages. Astrocytes were labeled with rabbit anti-GFAP (1:600; Dako, Carpinteria, CA). Neurons were labeled with mouse anti-NeuN (1:10,000; Millipore, Billerica, MA) or rabbit anti-βIII-tubulin (1:2000; Millipore). Schwann cells were labeled with rabbit anti-S100 (1:200; Sigma-Aldrich, St. Louis, MO).

For diaminobenzidine (DAB)-stained sections, samples were incubated for one hour with biotinylated horse anti-mouse antibody (1:80; Vector Laboratories, Burlingame, CA). Slides were rinsed and then incubated for 15 min with 5% hydrogen peroxide in methanol (fresh-frozen) or PBS (paraformaldehyde -immersed). Sections were rinsed and stored in ABC solution (Vector Laboratories) for 90 min. After rinsing, sections were labeled with 1 mg/ml DAB (Sigma-Aldrich) containing 10 mM imidazole and 0.03% hydrogen peroxide for ten minutes. Slides were rinsed, dehydrated, and coverslipped using Permount (Fisher Scientific) mounting medium.

For fluorescence-labeled sections, goat anti-mouse IgG1 (1:200), goat anti-mouse IgG2a (1:200), goat anti-mouse IgG (1:300), and/or goat anti-rabbit IgG conjugated to Alexa 568, Alexa 594, or Alexa 488 (Invitrogen, Carlsbad, CA) were applied to the sections for two hours at 37°C. Slides were rinsed and coverslips affixed with Vectashield (Vector Laboratories) mounting media supplemented with 4',6-diamidino-2-phenylindole (1 μg/ml, Sigma-Aldrich), and finally sealed with nail-polish.

### Image analysis and statistics

Images were taken using a Hamamatsu (model C4742-95) or PixeLink (model PL-A662) digital camera mounted on a wide-field fluorescence (Zeiss Axiophot) or on a bright-field (Nikon Diophot-TMD) microscope. Exposure times and magnification were kept constant among comparison groups. Quantification of grey-scale image files was performed using IPLab 3.6.5 software (Scanalytics, Inc. Fairfax, VA). Immunohistochemical staining was highlighted by setting the grey-level detection limits to a constant threshold, and the area of highlighted immunoreactivity was calculated as percent of total area (extent) and average signal intensity of the selected field. Tissues from hmSOD1 rats were compared directly to similar regions from wild-type rats that were immunostained together on the same slide to reduce technical variability.

For quantification of DAB-stained CD11b expression, between 20 and 30 total tissue sections of each spinal cord region were averaged together from each disease stage; between 13 and 23 total cross-section samples of nerves were averaged together per disease stage. The average area evaluated per region was 0.206 mm^2 ^for ventral horn, 0.258 mm^2 ^for ventral white matter, 0.213 mm^2 ^for dorsal horn, 0.121 mm^2 ^for ventral nerve root, 0.150 mm^2 ^for dorsal nerve root, and 0.209 mm^2 ^for sciatic nerve.

For quantification of GFAP and CD11b expression with dual immunofluorescence in the lumbar spinal cord, between 6 and 9 different ventral horn regions were averaged together per disease stage. The average area evaluated within the ventral horn was 0.145 mm^2^.

Extent and intensity levels were expressed as a fold-change with respect to averaged values measured in wild-type tissue. A Newman-Keuls test following an ANOVA analysis was used to determine significance among various disease stages from hmSOD1 tissues and wild-type tissues. A student t-test was performed to determine significance between hmSOD1 tissue from a specific disease stage and age-matched wild-type tissue. A *p*-value of less than 0.05 was considered significant.

## Results

### Clinical progression in hmSOD1 rat

**Figure 1:**
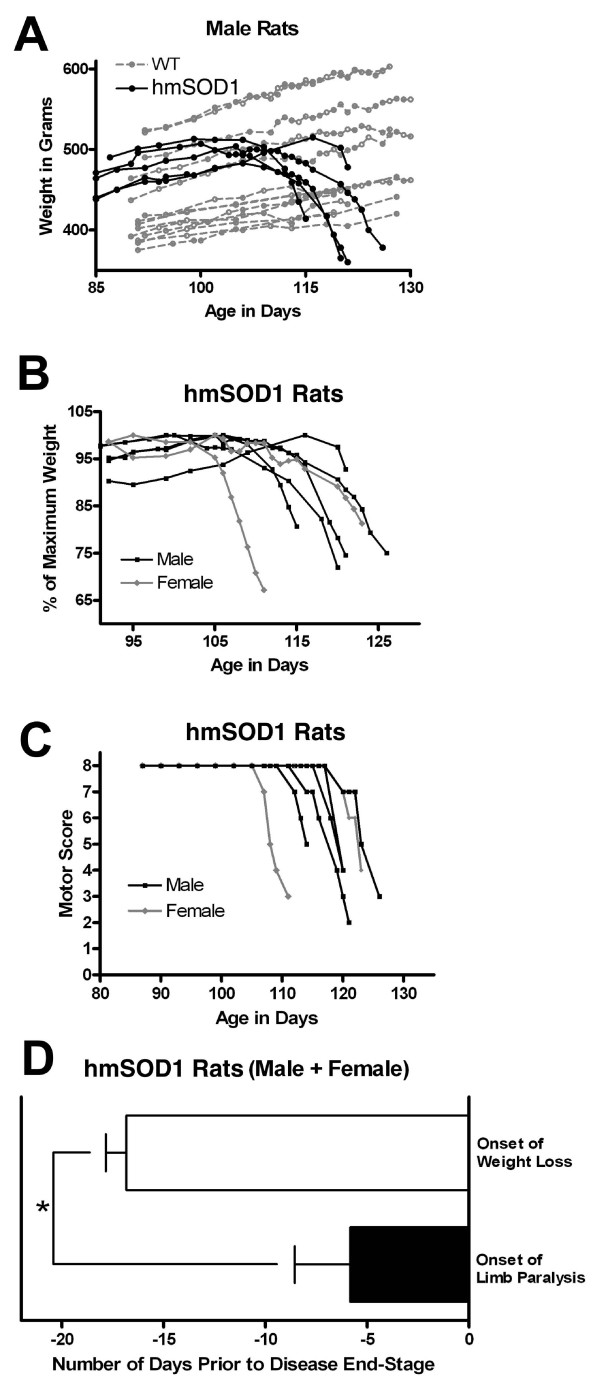
**Clinical onset and progression of disease**. Weight loss and limb motor abnormalities in hmSOD1 transgenic rats as a function of age. (A) Weight in grams of male hmSOD1 (black) and wild-type littermates (gray). (B) Weight expressed as percent of maximum recorded weight in male (black) and female (gray) hmSOD1 rats. (C) Limb paralysis motor score of male (black) and female (gray) hmSOD1 rats. See Table 1 for scoring criteria. (D) Average time between age of onset for weight loss and limb paralysis relative to disease end-stage in hmSOD1 rats. *, significantly different (*P *< 0.01, paired T test).

All hmSOD1 rats used in this study lost weight and had limb paralysis before 130 days of age while wild-type littermates continued to gain weight and never exhibited paresis or paralysis (Fig. 1A-C). Prior to 95 days of age, hmSOD1 rats gained weight at a similar rate as age- and sex-matched wild-type littermates (Fig. 1A). The onset of weight loss and development of limb paralysis occurred variably at ages 95-116 and 106-118 days, respectively (Fig. 1A-C). Limb paralysis presented either in a forelimb (2/7), hind limb (4/7), or simultaneously in a fore- and hind limb (1/7). Onset of weight loss preceded the first sign of limb paralysis by an average of 11 days (Fig. 1D). For this study, disease stages were based on weight change and detection of limb motor impairment as shown in Table 2.

### Change in CD11b expression in spinal cord

**Figure 2:**
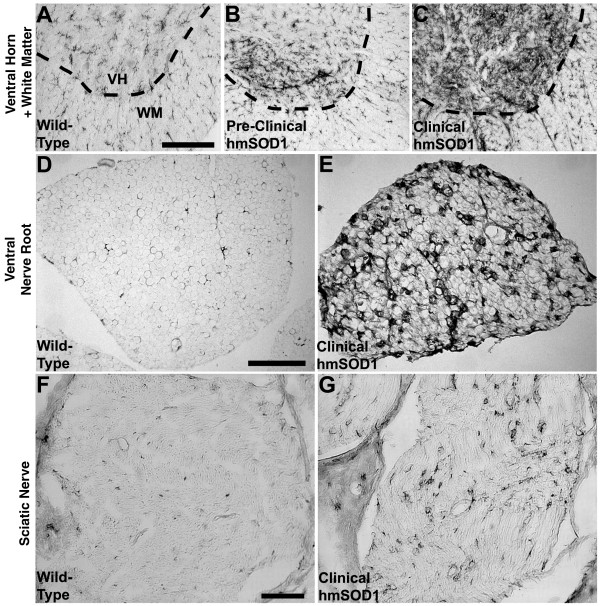
**CD11b-labeld microglia/macrophages in spinal cord and nerve**. Microglia or macrophages in the ventral spinal cord, ventral nerve roots, and sciatic nerve as assessed by CD11b histological expression. (A) CD11b-labeled microglia are evenly distributed in the ventral horn (VH, above dashed line) and in the ventral white matter (WM, below dashed line) of wild-type rats. (B) In pre-clinical hmSOD1 rats, focal increases in CD11b expression are often observed in the ventral horn. (C) After clinical onset, the extent of CD11b expression increases in ventral horn and to a lesser degree in the adjacent ventral white matter. Scale bar in panel A represents 200 μm for A-C. (D) There are very few CD11b-labeled resident macrophages in ventral nerve roots of wild-type rats. (E) After clinical onset in hmSOD1 rats, there is an increase in macrophages in the ventral nerve root. Scale bar in panel D represents 100 μm for D and E. (F) Very few CD11b-labeled resident macrophages are found in sciatic nerve of wild-type rats. (G) After clinical onset in hmSOD1 rats, there is an increase in macrophages in the sciatic nerve. Scale bar in panel F represents 100 μm for F and G.

**Figure 3:**
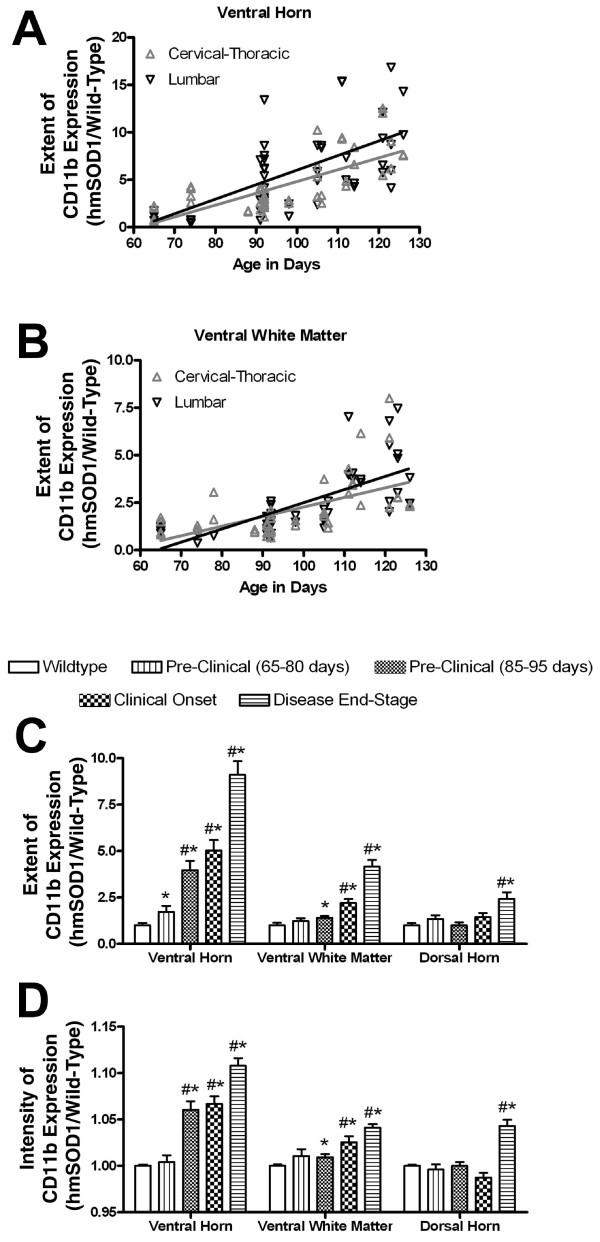
**Regional changes in CD11b expression in spinal cord**. Changes in the extent and intensity of DAB-stained CD11b expression in spinal cord of hmSOD1 rats as a function of age or disease stage. (A, B) The extent of CD11b expression relative to age-matched wild-type levels is expressed as a function of age. Linear regression slopes in the ventral horns of 0.12 ± 0.018 at the cervical-thoracic level and 0.15 ± 0.028 at the lumbar level (A). Linear regression slopes in the ventrolateral white matter are 0.05 ± 0.011 at the cervical-thoracic level and 0.069 ± 0.0107 at the lumbar level (B). (C, D) The average extent (C) and intensity (D) of CD11b expression relative to wild-type levels in spinal cord regions expressed as a function of disease stage. Bars represent average + SEM. #, significantly different compared to wild-type levels (*P *< 5 × 10^-5^, Neuman-Keuls test). *, significantly different compared to levels from only age-matched wild-type rats (*P *< 0.05, unpaired T test).

Microglia were assessed in spinal cords for changes in extent (occupied area) and intensity (average signal intensity) of CD11b expression. In wild-type littermates, microglia occupied 3.2% and 1.1% of the total tissue area within the ventral horn and ventral white matter, respectively, throughout the spinal cord. In hmSOD1 rats, the extent of CD11b expression increased in the ventral horn and white matter with age (Figs. 2B,C and 3A,B). In early pre-clinical hmSOD1 rat, there was a 72 +/- 32% increase in the extent of expression relative to wild-type tissue in the ventral horn with no significant change in the adjacent white matter region. In late pre-clinical hmSOD1 rat, there was a 297 +/- 50% increase in the ventral horn and a 40 +/- 10% increase in the ventral white matter (Figs. 2B and 3C). A significant increase in average tissue intensity of CD11b expression relative to wild-type tissue was also detected in the ventral horn (6.0 +/- 0.9%) in late pre-clinical hmSOD1 rat and in the ventral white matter (2.5 +/- 0.6%) during clinical onset (Fig. 3D). The extent and intensity of CD11b expression increased further as age and disease progressed (Fig. 2B,C and 3A-D). No changes in CD11b expression were measured in the dorsal horn until disease end-stage (Fig. 3C,D).

### Changes in CD11b expression in nerve root and sciatic nerve

**Figure 4:**
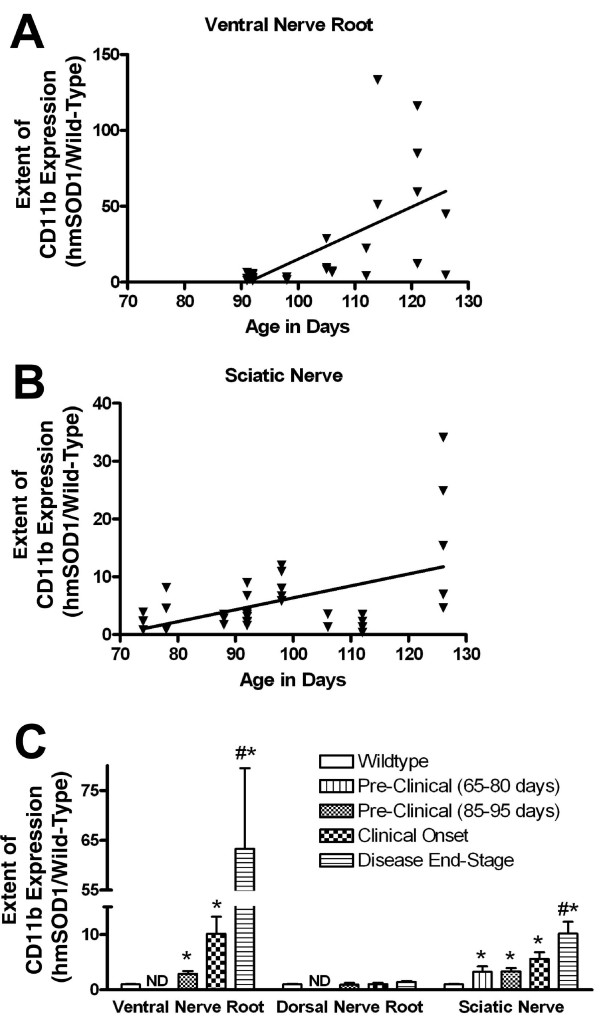
**Changes in CD11b expression in nerve roots and sciatic nerve**. Changes in the extent of DAB-stained CD11b expression in lumbar nerve roots and sciatic nerves of hmSOD1 rats. (A,B) The extent of CD11b expression relative to age-matched wild-type levels is expressed as a function of age. Linear regression slopes are 1.7 ± 0.45 in the ventral nerve roots (A) and 0.21 ± 0.063 in the sciatic nerve (B). (C) The average extent of CD11b expression relative to wild-type levels is expressed as a function of disease stage. Bars represent average + SEM. ND, not determined. #, significantly different compared to wild-type levels (*P *< 0.0005, Neuman-Keuls test). *, significantly different compared to levels from only age-matched wild-type rats (*P *< 0.05, unpaired T test).

Only a few CD11b-postive resident macrophages were observed in nerve roots (0.25% of tissue area) and sciatic nerves (0.58% of tissue area) from wild-type rats (Fig. 2D,F). In hmSOD1 rats, the extent of CD11b expression increased in the ventral nerve roots and sciatic nerves with age and disease progression (Fig. 2E,G and 4A-C). In late pre-clinical hmSOD1 rat, the extent of CD11b expression increased 189 +/- 55% in the ventral nerve root and 236% +/- 59% in the sciatic nerve relative to wild-type levels (Fig. 4C). No change in CD11b expression was measured in the dorsal nerve roots throughout the course of disease (Fig. 4C).

### Correlation of CD11b and GFAP expression in ventral horn

**Figure 5:**
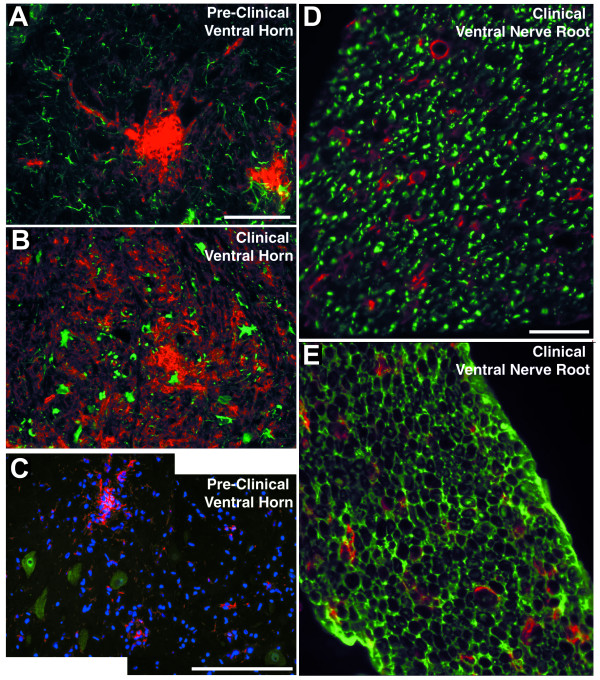
**Microglia/macrophages with neurons, astrocytes, and Schwann cells**. Dual immunofluorescence of microglia in the ventral horn and macrophages in the ventral nerve root of hmSOD1 rat. (A,B) GFAP-labeled astrocytes (green) and CD11b-labeled microglia (red) in a ventral horn at pre-clinical stage (A) and after clinical onset (B). Scale bar in panel A represents 100 μm for A and B. (C) Composite image showing Iba1-labeled microglia (red) near NeuN-labeled motor neurons (green) in a pre-clinical rat. Scale bar in panel C represents 200 μm. (D,E) CD11b-labeled macrophages (red) relative to βIII-tubulin-labeled axons (D, green) or S100-labeled Schwann cells in ventral nerve root after clinical onset (E, green). Scale bar in D represents 100 μm for D and E.

**Figure 6:**
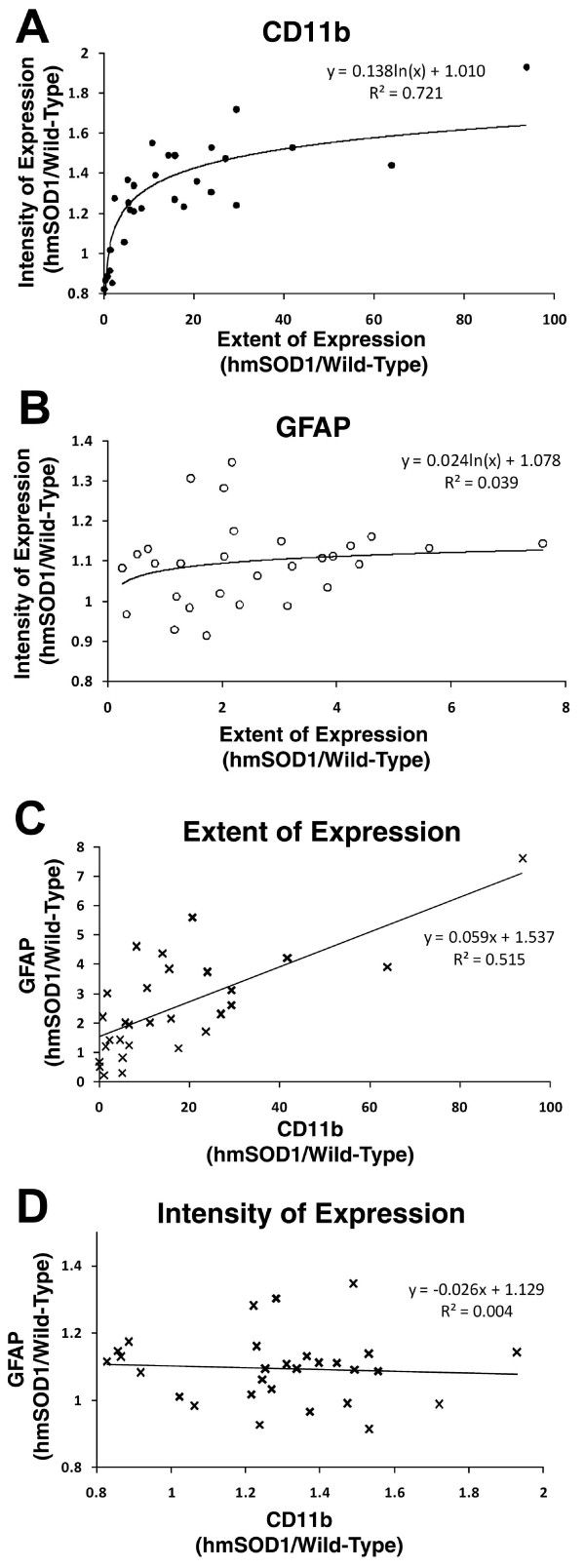
**Correlation between CD11b and GFAP expression in ventral horn**. Changes in GFAP and CD11b in lumbar ventral horn detected with dual immunofluorescence throughout disease progression. (A,B) The extent relative to intensity of CD11b (A) and GFAP (B) expression. (C) The extent of expression of CD11b relative to GFAP expression. (D) The tissue intensity of CD11b relative to GFAP expression.

GFAP-labeled astrocytes in the ventral horn of hmSOD1 rats were hypertrophic during later clinical stages of disease development, but not pre-clinically (Fig. 5A,B). While increased extent and intensity of CD11b expression revealed a strong positive logarithmic correlation (r^2 ^= 0.72) throughout disease progression, the extent and intensity of GFAP expression did not correlate (Fig. 6A,B). There was a moderate linear correlation (r^2 ^= 0.52) between increased extent of CD11b and GFAP expression (Fig. 6C), but not between tissue intensity of CD11b and GFAP (Fig. 6D).

### Microglia aggregate in the ventral horns prior to clinical onset

**Figure 7:**
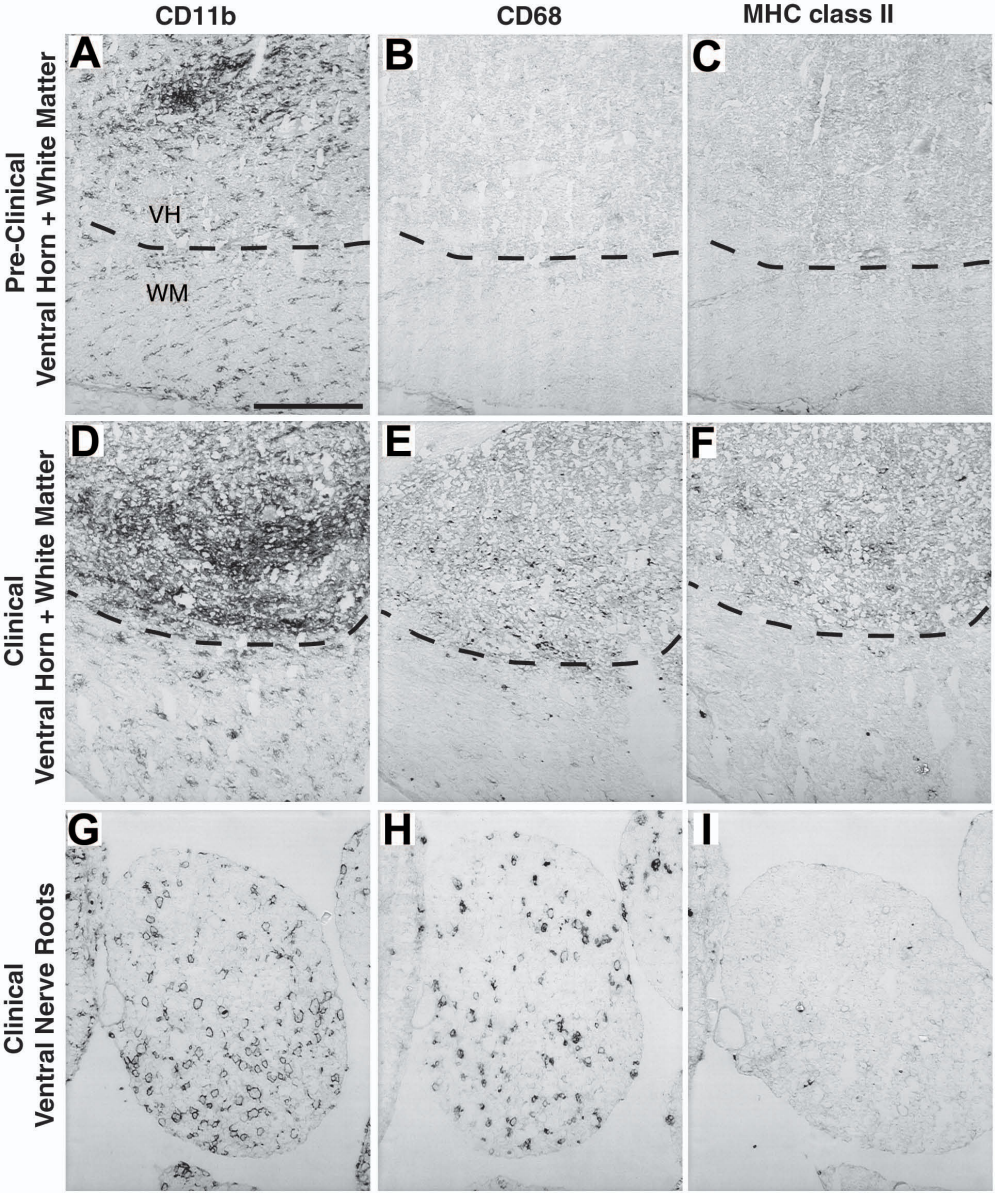
**Expression of CD11b, CD68, and MHC class II in spinal cord and ventral nerve root**. Expression of CD11b, CD68, and MHC II in adjacent sections of ventral spinal cord and ventral nerve root of hmSOD1 rats. (A-C) In pre-clinical stage ventral horn, a cluster of CD11b-labeled microglia (A) in the ventral horn (VH, above dashed line) does not co-localize with CD68 (B) or MHC II (C). (D-F) In the ventral horn after clinical onset, CD11b expression (D) is more widespread and both CD68 (E) and MHC II (F) expression are detected. (G-I) In ventral nerve root after clinical onset, both CD11b- (G) and CD68-labeled (H) cells are increased, but MHC II-labeled cells (I) remain sparse. Scale bar in panel A represents 200 μm for all panels.

Microglia in the ventral horns of pre-clinical hmSOD1 rats were typically found in focal aggregates that were identified with CD11b (Fig. 2B and 5A) or Iba1 (Fig. 5C) markers. GFAP-labeled astrocytes did not cluster together in the ventral horns early in disease (Fig. 5A) and remained relatively dispersed at disease end-stage (Fig. 5B). The intracellular labeling of Iba1 and nuclear counterstain revealed a dense clustering pattern for the microglia (Fig. 5C). These early focal clusters of microglia were often near, but rarely juxtaposed and never observed fully encapsulating NeuN-labeled motor neurons (Fig. 5C). Activation markers CD68 and MHC class II were observed in ventral horns of hmSOD1 rats after clinical onset (Fig. 7D-F), but were not remarkably expressed in pre-clinical rats even at sites of the focally elevated CD11b expression (Fig. 7A-C).

### Macrophages in nerve root and sciatic nerve

The CD11b-labeled macrophages seen in ventral nerve root and sciatic nerve from hmSOD1 rats typically formed ring-like morphologies in pre-clinical hmSOD1 rats through to disease end-stage (Figs. 2E,G, 5D,E, and 7G). Co-labeling of CD11b and βIII-tubulin in ventral nerve after clinical onset revealed many macrophages to be partially encompassing individual nerve fibers or forming complete ring-shapes at sites devoid of a βIII-tubulin-labeled axon (Fig. 5D). Co-labeling of CD11b and S100 showed the overall tissue architecture of Schwann cells remained relatively unperturbed at end-stage disease, while the macrophages were often juxtaposed between them and the axon (Fig. 5E). The nerve macrophages in hmSOD1 rats expressed the macrophage marker CD68, but seldom expressed MHC class II (Figs. 7G,I).

## Discussion

It is clear that microglia and macrophages are involved in the pathological processes of ALS and in the hmSOD1 transgenic animal models of the disease. What is uncertain is their role in pathogenesis and during the evolving pathophysiological process. Further understanding of their changes with respect to disease development is essential and may provide insight into their participation in motor neuron death. In this study, we evaluated spinal microglia and macrophages in the PNS before and after clinical manifestation in the transgenic hmSOD1 (G93A) rat based primarily on the changes in tissue expression of CD11b. This increasingly used transgenic animal model of ALS is relatively new and has not been as well characterized with regard to neuroinflammation and glial activation as the murine transgenic model. We show that microglia/macrophages are active early in the disease process both in the ventral horn where the soma of lower motor neurons reside and along the path of their axons in the adjacent ventrolateral white matter region and peripheral nerve.

The hmSOD1 rats used in this study were categorized as being pre-clinical, at clinical onset, or near disease end-stage. Since a loss in weight gain preceded the onset of limb paralysis, which has also been documented in a separate transgenic line of hmSOD1 rat [27], reduced rate of weight change was used to distinguish between pre-clinical and clinical onset. Weight loss is also an early clinical presentation in the murine model and precedes more sensitive detections of motor dysfunction such as the paw grip endurance test [28]. The average age of pre-clinical rats used in this analysis was more than two weeks earlier than that the average age of clinical onset. Limb paralysis was not yet present in rats classified as at clinical onset.

In the ventral horn of pre-clinical rats, an enhancement of CD11b tissue expression and the formation of microglia in focal aggregates were observed. Atypical clusters of microglia have been described previously as the cells fusing together to form multinucleated giant cells of the Langhans type following clinical onset [5]. It is difficult to prove that the microglia have fused and formed giant cells based on immunohistochemical analysis alone, but the cytological appearance of irregular plasmalemma borders and haphazard arrangement of nuclei suggests that they are individual cells aggregating rather than fused giant cells. The microglia may be targeting degenerating neurons and in a process of neuronophagia in which phagocytes engulf a dying neuron. However, we only found occasional neurons partially encompassed by these formations at the pre-clinical stage, and did not find neurons fully encircled. It is plausible that at the time of neuronophagia the neuron had degenerated to an extent that it no longer was identifiable with a marker such as NeuN. Yet, it is also important to note that the activation markers CD68 and MHC class II, which are normally upregulated in microglia during phagocytosis [29-32], did not co-localize with these formations at pre-clinical stage of disease. Alternatively, microglia may not be responding to a dying neuron but rather an unidentified extra-neuronal stimulus such as aggregates of mutant SOD1 protein or paracrine signals from cytokines or other secreted molecules. These clusters of microglia may then secondarily cause damage to nearby motor neurons. Increased mRNA for IL-1β, IL-12, MIPα,β, TGF-β, and TNF-α have been reported in the hmSOD1 rat at pre-clinical stage of disease [20]. These changes in cytokine expression are likely associated with the function of the aggregating microglia. Further analysis is needed to determine what role these clusters of microglia have in the neurodegeneration process.

Pre-clinical rats also showed an increase of microglia in the ventrolateral white matter, where lower motor neuron axons are known to cross, and an increase in macrophages in the ventral (motor) nerve root and in the sciatic nerve. No macrophage accumulation occurred in the dorsal (sensory) nerve root indicating a specific association of macrophage changes with lower motor neuron axons. Dal Canto and Gurney identified macrophages in the PNS of transgenic hmSOD1 mice using electron microscopy when the animal model was initially developed [25], but more descriptive data on this phenomenon has been lacking to date. Resident macrophages comprise less than five percent of the cell population of the peripheral nerve [33]. They express several macrophage antigens including CD11b, CD68, and MHC class II [34]. The accumulated macrophages in the hmSOD1 peripheral nerve expressed CD11b and CD68, but only few of these cells expressed MHC class II. This is similar to what is observed following nerve injury model [34,35]. The macrophages in the hmSOD1 nerve appeared as rings formed around individual axons, which is also similar to the response after nerve injury [35,36]. In the murine hmSOD1 model, activation of Schwann cells in the peripheral nerve prior to clinical onset has been reported based on increased GFAP expression [37]. This activation of Schwann cells in addition to our observation of increased macrophages in the nerves of hmSOD1 rats might indicate a response to Wallerian degeneration associated with dying-back of motor axons that has been described previously in the mouse model [24]. Although increased macrophages were detected pre-clinically in both the ventral nerve root and more distally at the sciatic nerve, it would be important to know when or if macrophages accumulate closer to the neuromuscular junction in nerves distal to the branching of the sciatic nerve. It cannot be ruled out that these macrophages are responding to an unidentified stimulus or that they contribute to axonal damage. Further investigation is warranted.

As rats progressed to clinical onset and even further to disease end-stage, the accumulation of microglia/macrophages increased further in the ventral horn, the adjacent white matter region, ventral nerve root, and sciatic nerve. Within the ventral horn, hypertrophic astrocytes were now present. This timing of increased astrocyte activity supports previous findings in the murine model [3,37], although Keller and colleagues observed temporary increases at earlier stages using live imaging analysis. Surprisingly, the increase in the extent or intensity of astrocytic GFAP tissue expression did not have a strong correlation with increases in microglial CD11b expression within the same tissue samples. We propose that microglial activation based on CD11b expression, not astrocytic activation, is a superior histological marker for disease progression in the rat transgenic model of ALS.

Microglial activation markers MHC class II and CD68 appeared in a subpopulation of cells after clinical onset in the ventral horn as the extent and intensity of CD11b tissue expression continued to increase. For MHC class II, this stage of detection is similar to its onset in the murine model [3]. Several studies reported CD68 expression in the ventral horn in late stages of disease development [23,38,39]. Few CD68-positive cells were detected at pre-clinical stages in the murine model that had become widespread throughout the ventral horn at later stages [23]. Also in the murine model, mRNA levels of CD68 have been shown to be elevated just prior to clinical onset and then increased further as disease progressed [21,23]. We did not find remarkable CD68 expression until after clinical onset in the rat model and it never was as widespread relative to the CD11b expression. The expression pattern we observed was more punctuate, likely representing lysosomal and not plasma membrane expression, relative to the murine model. The discrepancies in the onset of CD68 expression and pattern of cellular expression between the rat and mouse model might have to do with the different antibodies used, which are species specific and may bind different isoforms or glycosylation states of CD68.

In hmSOD1 mice, pre-clinical administration of immunosuppressive drugs prolonged survival [10-12], while a separate study reported that continually ablated proliferating microglia in lumbar spinal cord beginning close to clinical onset resulted in decreased microgliosis without protection of motor neurons [22]. Interestingly, modulating microglia activity by eliminating functional T lymphocytes in the murine hmSOD1 model of ALS decreased mRNA levels of CD68 and increased levels of TNF-α and NOX2, which lead to a shorter survival duration without any change in time of onset [40,41]. Although CD11b expression was increased prior to clinical onset in our study, other microglia activation markers (CD68 and MHC class II) and astrogliosis were detected in the ventral horn after clinical onset. Taken together, we suggest that the immunological activity occurring prior to clinical onset is involved in the induction of motor neuron degeneration, whereas the activity observed after clinical onset is more involved with other cell functions such as clearing the debris of degenerated neurons.

## Conclusion

In summary, this immunohistochemical investigation of the rat transgenic model of ALS describes the early accumulation of microglia occurring in the ventral horn and in adjacent white matter, and macrophages in the ventral nerve root and sciatic nerve, corresponding with the path of the soma and axon of lower motor neurons. The macrophage activity occurring in the nerves needs to be taking into account in studies involving immunomodulation in transgenic animal models of ALS. We propose that the pathophysiological immune activity is different before and after clinical onset of disease. Further understanding of the microglia/macrophage activity occurring prior to clinical onset may shed light on the unresolved mechanism of neurodegeneration and lead to more targeted immunomodulation therapies that are effective in stopping neuron loss in ALS.

## Competing interests

The authors declare that they have no competing interests.

## Authors' contributions

Design of studies DJG, WFH, BTH. Animal handling, processing of tissue samples, and imaging/quantification DJG. Writing/reviewing of manuscript DJG, WFH, BTH. All authors have read and approved the final manuscript.
